# Impact of obesity on clinical outcomes in patients treated with ceftobiprole: results from Phase 3 clinical trials

**DOI:** 10.1093/jac/dkaf096

**Published:** 2025-03-28

**Authors:** Thomas L Holland, Andrew F Shorr, J Scott Overcash, Marc Engelhardt, Mark Jones, Daniel Ionescu, Karine Litherland, Mikael Saulay, Vance G Fowler

**Affiliations:** Division of Infectious Diseases, Duke University School of Medicine, Durham, NC, USA; Duke Clinical Research Institute, Duke University School of Medicine, Durham, NC, USA; Section of Pulmonary, Critical Care, and Respiratory Services, MedStar Washington Hospital Center, Washington, District of Columbia, USA; Velocity Clinical Research, San Diego, CA, USA; Basilea Pharmaceutica International Ltd, Allschwil, Switzerland; Basilea Pharmaceutica International Ltd, Allschwil, Switzerland; Basilea Pharmaceutica International Ltd, Allschwil, Switzerland; Basilea Pharmaceutica International Ltd, Allschwil, Switzerland; Basilea Pharmaceutica International Ltd, Allschwil, Switzerland; Division of Infectious Diseases, Duke University School of Medicine, Durham, NC, USA; Duke Clinical Research Institute, Duke University School of Medicine, Durham, NC, USA

## Abstract

**Background:**

Ceftobiprole was non-inferior to comparators for the treatment of *Staphylococcus aureus* bloodstream infection (bacteraemia) (SAB), acute bacterial skin and skin structure infection (ABSSSI), and community-acquired bacterial pneumonia (CABP), leading to regulatory approval for these indications. Whether dosing should be modified for patients with obesity is unknown.

**Objectives:**

This *post hoc* analysis evaluated the relationship of obesity and clinical outcomes in patients treated with ceftobiprole for SAB, ABSSSI or CABP.

**Methods:**

Efficacy and safety outcomes were assessed based on BMI from three registrational clinical trials that evaluated ceftobiprole against comparators.

**Results:**

Overall, 1641 patients were included from the three Phase 3 clinical trials (802 ceftobiprole; 839 comparators). When stratifying by BMI, ceftobiprole had similar outcomes to the overall ceftobiprole population (80.4%) including patients with obesity (BMI = 30–40 kg/m^2^) (81.7%). Severe obesity (BMI ≥ 40 kg/m^2^) was associated with decreased clinical cure rates overall (68.2%) compared with the overall ceftobiprole population, and this was especially noted in the clinically evaluable patient population with CABP receiving ceftobiprole (66.7% in severe obesity versus 86.6% overall). This was also seen in the comparator group (33.3% in severe obesity versus 87.4% overall). However, the number of patients with severe obesity was low in the CABP trial. The safety profile was similar between treatment groups in all studies and not influenced by BMI.

**Conclusions:**

This analysis further supports the efficacy and safety of ceftobiprole at current recommended doses in obese patients with SAB, ABSSSI or CABP.

## Introduction

Worldwide, adult obesity has more than doubled since 1990. Currently, 43% of adults are considered overweight (BMI ≥ 25 kg/m^2^) and 16% are obese (BMI ≥ 30 kg/m^2^).^1^ Obese patients may have physiological alterations that influence the pharmacokinetics (PK) of drugs,^2^ resulting in either toxicity or therapeutic failure. For example, obesity is a risk factor for the development of nephrotoxicity with vancomycin,^3^ and obese patients treated with daptomycin have higher rates of creatine phosphokinase elevations and treatment discontinuation.^4^ In recent years, additional studies of antimicrobial PK/pharmacodynamics (PK/PD) parameters, extended infusion administration, and drug levels in critically ill patients have made it possible to provide more appropriate dosing for obese patients.^2,5,6^

Ceftobiprole is an advanced-generation cephalosporin with bactericidal activity against Gram-positive and Gram-negative bacteria, including MRSA and MSSA. In the USA, ceftobiprole is approved for adult patients with *Staphylococcus aureus* bloodstream infection (bacteraemia) (SAB), including those with right-sided infective endocarditis, adult patients with acute bacterial skin and skin structure infection (ABSSSI), and adult and paediatric patients (3 months to less than 18 years old) with community-acquired bacterial pneumonia (CABP).^7^ Additional indications outside the USA include hospital-acquired pneumonia, excluding ventilator-associated pneumonia.^8^ A previous Phase 1 study assessed the PK, PD, safety and tolerability of ceftobiprole in severely obese (BMI ≥ 40 kg/m^2^) and non-obese (BMI = 18–30 kg/m^2^) healthy adult (aged ≥18 years) subjects. Following a single infusion of ceftobiprole 500 mg, the volume of distribution and clearance were higher, and drug exposures (*C*_max_ and AUC_∞_) were ∼17% lower in severely obese adults compared with non-obese individuals. However, because percent time of free drug above the MIC (%*fT*_>MIC_; the PD parameter associated with efficacy of ceftobiprole) values were similar between the two groups, it was concluded that clinicians likely did not need to adjust the dose of ceftobiprole for severely obese patients.^9^ In the current report, we compared the clinical outcomes of patients from three large Phase 3 clinical trials who were treated with either ceftobiprole or comparator therapy based upon their BMI levels.

## Materials and methods

### Study design and patient population

This was a *post hoc* analysis of three randomized, double-blind, multicentre, Phase 3 trials in patients hospitalized with SAB (NCT03138733), an ABSSSI (NCT03137173) or CABP (NCT00326287). The full study methods of the three trials have been previously described and are summarized in Table S1 (available as Supplementary data at *JAC* Online).^10–12^ For all studies, institutional review board/ethics committee approval of the protocol was obtained, and all participants provided signed written informed consent before enrolment. This analysis included subgroups of patients with BMIs of <25, 25 to <30, 30 to <40 (obese) and ≥40 kg/m^2^ (severely obese) at enrolment, based on standard definitions by the National Institutes of Health and WHO classification of weight status.^1,13^ Because the number of patients with a BMI of ≥40 was small, additional *post hoc* analyses were conducted with thresholds of BMI < 30/≥30 kg/m^2^ and <35/≥35 kg/m^2^ to further assess obesity on clinical outcomes. For all efficacy endpoints, analyses were conducted based on the primary endpoint population of the ITT population (ABSSSI and CABP),^11,12^ modified ITT (MITT) population (SAB)^10^ and clinically evaluable (CE) population (CABP).^12^ Definitions of analysis populations are provided in the Supplementary data. Safety and tolerability were evaluated by assessment of adverse events (AEs) in the safety population (defined as all randomized patients who received at least one dose of the study drug) in the standard BMI definition thresholds only.

### Post hoc and statistical analysis

Statistical methods for the key primary and secondary endpoints and safety assessments, including sample size calculations and non-inferiority, have been published.^10–12^ Exploratory subgroup analyses assessing the impact of BMI on clinical outcomes were performed for the pooled primary endpoint populations. The BMI subgroups were defined programmatically from patients’ baseline information. Data were summarized descriptively by BMI groups using the primary analysis population from each study. For the pooled efficacy analysis, a two-sided 95% CI for the treatment difference was computed using normal approximation.

## Results

### Patient disposition and baseline characteristics

Overall, 1641 patients were included in the evaluation from the ITT or MITT populations of the SAB (23.5%), ABSSSI (41.3%) and CABP (35.2%) trials (Table 1). Of these, 20.7% (341/1641) were considered obese and 3% (49/1641) were considered severely obese (Table 1). Most obese and severely obese patients were in the ABSSSI trial [12.5% (205/1641)], followed by the SAB trial [6% (99/1641)]. Across the ITT or MITT populations in this evaluation, 802 patients were randomized to ceftobiprole (SAB, *n* = 187; ABSSSI, *n* = 334; CABP, *n* = 281) and 839 were randomized to comparator drugs (SAB, *n* = 198; ABSSSI, *n* = 344; CABP, *n* = 297). Key baseline characteristics of each trial are presented in Table 2. Baseline characteristics were mostly similar except there was a higher percentage of patients with diabetes (35.1%) and renal dysfunction (CL_CR_ < 50 mL/min) in the SAB trial, signifying the risk factors and severity of the infection. A BMI of >30 kg/m^2^ was associated with higher rates of diabetes mellitus across all three trials. Furthermore, patients with severe obesity (BMI ≥ 40 kg/m^2^) were more likely to have augmented renal clearance (ARC) (CL_CR_ > 130 mL/min) compared with the other groups.

**Table 1.** Number of patients in each BMI group from the SAB, ABSSSI and CABP Phase 3 trials based on primary efficacy endpoints (ITT population)

	SAB^a^		ABSSSI		CABP		SAB + ABSSSI + CABP		Total
BMI group (kg/m^2^)	Ceftobiprole	Daptomycin	Ceftobiprole	Vancomycin + aztreonam	Ceftobiprole	Ceftriaxone ± linezolid	Ceftobiprole	Comparator	
<25	68	65	125	128	152	169	345	362	707
25 to <30	73	80	103	117	84	87	260	284	544
30 to <40	41	44	94	87	40	35	175	166	341
≥40	5	9	12	12	5	6	22	27	49
Total	187	198	334	344	281	297	802	839	1641

^a^MITT population.

**Table 2.** Demographic and baseline characteristics based on BMI (kg/m^2^) of patients from the SAB, ABSSSI and CABP Phase 3 studies (ITT population)

Characteristics	SAB^a^				ABSSSI				CABP				Total			
	BMI (kg/m^2^)															
	< 25 (*N* = 133)	25 to <30 (*N* = 153)	30 to < 40 (*N* = 85)	≥40 (*N* = 14)	< 25 (*N* = 253)	25 to <30 (*N* = 253)	30 to <40 (*N* = 181)	≥40 (*N* = 24)	<25 (*N* = 321)	25 to < 30 (*N* = 171)	30 to <40 *N* = 75)	≥40 (*N* = 11)	<25 (*N* = 707)	25 to <30 (*N* = 544)	30 to <40 (*N* = 341)	≥40 (*N* = 49)
Age (years)																
Median	56.0	55.0	61.0	61.0	44.0	49.0	54.0	50.0	52.0	61.0	60.0	55.0	51.0	53.0	56.0	56.0
Min–max	1 9–90	19–85	33–91	55–67	18–79	23–87	21–89	30–86	18–90	18–94	22–86	29–86	18–90	18–94	21–91	2 9–86
Gender, *n* (%)																
Female	34 (25.6)	44 (28.8)	31 (36.5)	10 (71.4)	89 (35.2)	84 (38.2)	90 (49.7)	17 (70.8)	133 (41.4)	66 (38.6)	40 (53.3)	5 (45.5)	256 (36.2)	194 (35.7)	161 (47.2)	32 (65.3)
Male	99 (74.4)	109 (71.2)	54 (63.5)	4 (28.6)	164 (64.8)	136 (61.8)	91 (50.3)	7 (29.2)	188 (58.6)	105 (61.4)	35 (46.7)	6 (54.5)	451 (63.8)	350 (64.3)	180 (52.8)	17 (34.7)
Geographic regions, *n* (%)																
Europe	122 (91.7)	143 (93.5)	80 (94.1)	13 (92.9)	74 (29.2)	78 (35.5)	95 (52.5)	14 (58.3)	136 (42.4)	77 (45.0)	42 (56.0)	5 (45.5)	332 (47.0)	298 (54.8)	217 (63.6)	32 (65.3)
North America	3 (2.3)	5 (3.3)	1 (1.2)	1 (7.1)	179 (70.8)	142 (64.5)	86 (47.5)	10 (41.7)	28 (8.7)	27 (15.8)	20 (26.7)	3 (27.3)	210 (29.7)	174 (32.0)	107 (31.4)	14 (28.6)
Other	8 (6.0)	5 (3.3)	4 (4.7)	0	0	0	0	0	157 (48.9)	67 (39.2)	13 (17.3)	3 (27.3)	165 (23.3)	72 (13.2)	17 (5.0)	3 (6.1)
Diabetes mellitus, *n* (%)	34 (25.6)	50 (32.7)	43 (50.6)	7 (50.0)	6 (2.4)	24 (10.9)	37 (20.4)	10 (41.7)	26 (8.1)	30 (17.5)	14 (18.7)	3 (27.3)	66 (9.3)	104 (19.1)	94 (27.6)	20 (40.8)
Baseline CL_CR_ (mL/min), *n* (%)																
>130	21 (15.8)	41 (26.8)	23 (27.1)	9 (64.3)	70 (27.7)	88 (40.0)	78 (43.1)	16 (66.7)	24 (7.5)	23 (13.5)	22 (29.3)	6 (54.5)	115 (16.3)	152 (27.9)	123 (36.1)	31 (63.3)
>80 to <130	53 (39.8)	59 (38.6)	30 (35.3)	2 (14.3)	133 (52.6)	102 (46.4)	77 (42.5)	5 (20.8)	124 (38.6)	73 (42.7)	29 (38.7)	3 (27.3)	310 (43.8)	234 (43.0)	136 (39.9)	10 (20.4)
≤50 to ≤80	25 (18.8)	27 (17.6)	15 (17.6)	1 (7.1)	40 (15.8)	25 (11.4)	20 (11.0)	2 (8.3)	107 (33.3)	45 (26.3)	18 (24.0)	1 (9.1)	172 (24.3)	97 (17.8)	53 (15.5)	4 (8.2)
≤30 to <50	9 (6.8)	5 (3.3)	3 (3.5)	1 (7.1)	9 (3.6)	5 (2.3)	6 (3.3)	1 (4.2)	37 (11.5)	24 (14.0)	4 (4.3)	0	55 (7.8)	34 (6.3)	13 (3.8)	2 (4.1)
<30	6 (4.5)	3 (2.0)	4 (4.7)	0	1 (0.4)	0	0	0	10 (3.1)	1 (0.6)	0	0	17 (2.4)	4 (0.7)	4 (1.2)	0
Chronic dialysis	19 (14.3)	18 (11.8)	19 (14.3)	1 (7.1)	0	0	0	0	0	0	0	0	19 (2.7)	18 (3.3)	10 (2.9)	1 (2.0)

CL_CR_ based on the Cockcroft–Gault formula.

^a^MITT population.

### Efficacy/clinical response

**Figure 1.**
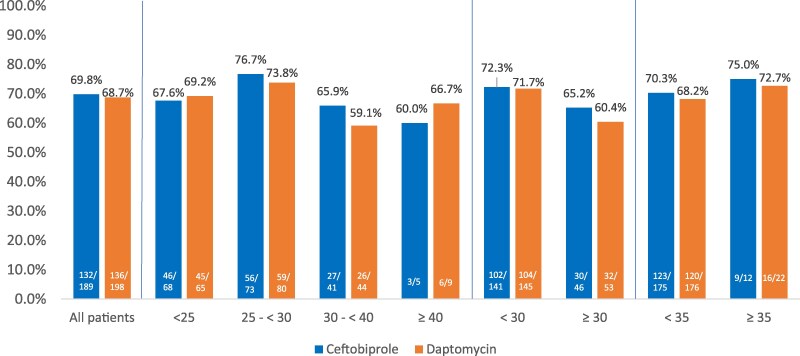
Overall success at post-therapy evaluation visit by BMI categories (kg/m^2^) in SAB trial (MITT population).

**Figure 2.**
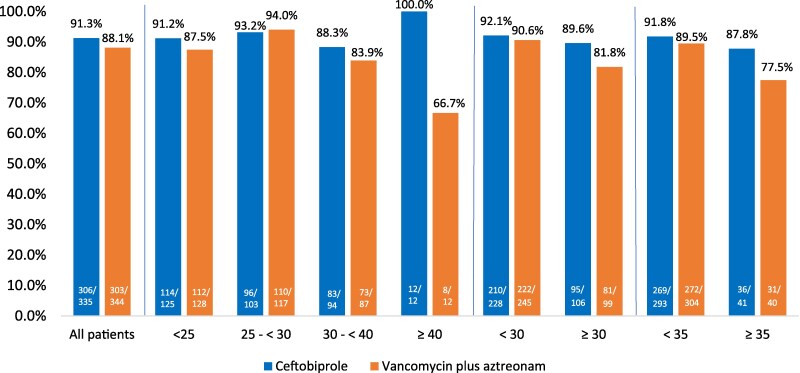
Early clinical response 48–72 h after start of treatment by BMI categories (kg/m^2^) in ABSSSI trial (ITT population).

**Figure 3.**
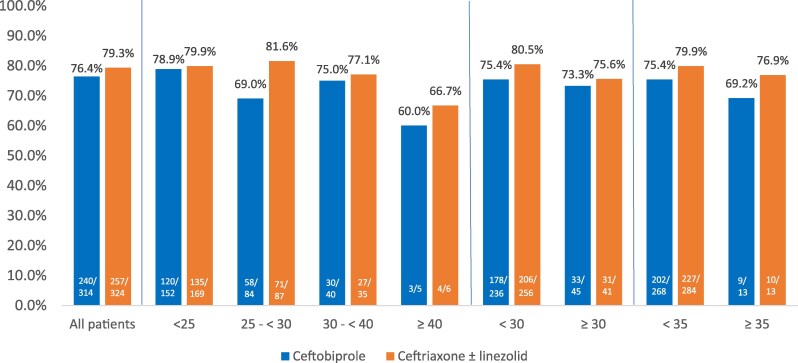
Clinical cure rates at TOC visit by BMI categories (kg/m^2^) in CABP trial (ITT population).

**Figure 4.**
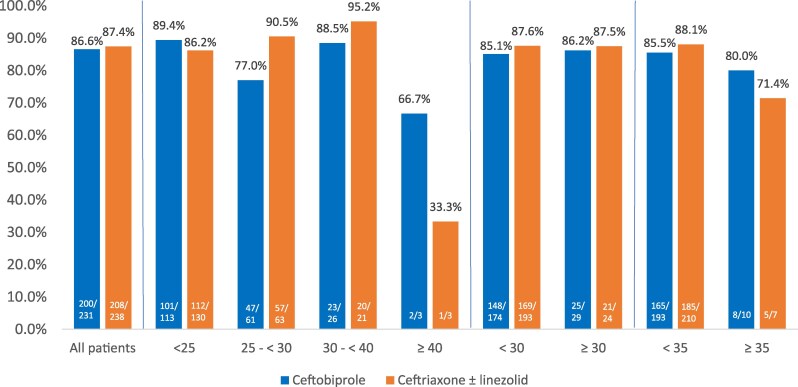
Clinical cure rates at TOC visit by BMI categories (kg/m^2^) in CABP trial (CE population). CE population: all treated patients with a diagnosis of CAP, unless the duration of study drug therapy was <48 h or <80% of the intended dose, cure took place within 5 days, or if other prespecified exclusion criteria applied.

**Figure 5.**
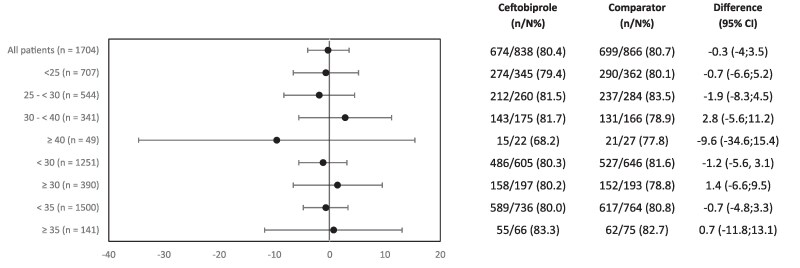
Forest plot of proportion for overall success/clinical cure rates at PTE/TOC by BMI categories (kg/m^2^) in the SAB, ABSSSI and CABP Phase 3 trials (primary endpoint populations pooled). There were 1641 patients included in the analysis. The number 1704 indicates the total number of patients across all three trials. There were 63 patients with missing BMI information across the three trials.

The SAB and ABBSSI trials both involved comparators that are dosed based on weight (daptomycin and vancomycin, respectively). In all three trials, the treatment differences for the primary endpoint populations based on BMI were similar between ceftobiprole and comparators (Figures 1–4). Clinical cure rates (defined in Table 3) were broadly similar across treatment groups and BMI-stratified subgroups in each individual study. When all the patients were combined from each study, no difference in outcomes based on BMI was observed. This was further demonstrated when evaluating outcomes across different BMI thresholds (<30/≥30 kg/m^2^ and <35/≥35 kg/m^2^) (Figure 5). In other words, outcomes for ceftobiprole-treated patients did not vary as a function of BMI.

**Table 3.** Definitions of clinical efficacy based on primary endpoints for the SAB, ABSSSI and CABP Phase 3 trials

SAB
For the SAB trial, treatment success was assessed at 70 days after randomization and was defined as survival, symptom improvement, SAB clearance, absence of new SAB-related complications, and no use of other potentially effective antibiotics.
ABBSSI
For the ABSSSI study, early clinical response was assessed at 48–72 h after the start of treatment, and success was defined as meeting all of the following: ≥20% reduction from baseline in the area of the primary lesion, survival for ≥72 h from initiation of treatment, no use of concomitant systemic or topical antibacterials on the primary lesion and no unplanned surgical procedures for the ABSSSI after the start of treatment.
CABP
For the CABP trial, clinical cure was assessed at the TOC visit and was defined by either resolution of signs and symptoms of infection (return to pre-infection status) or sufficient improvement such that no further antibacterial therapy was necessary and improvement or no adverse changes in findings on the chest radiograph.

### SAB trial

The ceftobiprole dose in the SAB trial was 500 mg every 6 h for the first 8 days as a 2 h regimen followed by every 8 h thereafter. The rationale for this higher dosing regimen was to achieve rapid and early killing of *S. aureus,* in contrast to prior experience with daptomycin, in which the median time to clearance was 8–9 days.^10,14^ Overall success at 70 days after randomization were similar between the different BMI categories (Figure 1). The overall success rate of ceftobiprole at the post-treatment evaluation (PTE) was 68.8%. The rates of success for ceftobiprole were similar to the overall results at BMIs of <25 (67.6%), 25 to <30 (76.7%), 30 to <40 (65.9%) and ≥40 kg/m^2^ (60%). Results were also similar at the BMI thresholds of <30 (72.3%), ≥30 (65.2%), <35 (70.3%) and ≥35 (75%). Of note, only 11.1% of patients (*n* = 22) in the SAB trial received >7 mg/kg doses of daptomycin, and daptomycin dose was not associated with treatment success.^10^

### ABSSSI trial

Early clinical response at 48–72 h was similar across the treatment groups in the different BMI categories. The clinical response rates with ceftobiprole were 91.2% at BMIs of <25, 93.2% at BMIs of 25 to <30, 88.3% at BMIs of 30 to <40% and 100% at BMIs of ≥40 kg/m^2^ and were comparable to the overall ceftobiprole response rate of 91.3%. Ceftobiprole maintained high response rates across all BMI categories including at the additional BMI thresholds of <30 (92.1%), ≥30 (89.6%), <35 (91.8%) and ≥35 (87.8%). In contrast, treatment success among patients randomized to vancomycin + aztreonam was lower (66.7% versus 88.1% overall) in patients with a BMI of ≥40 (Figure 2). Of note, in patients with severe obesity, ceftobiprole (*n* = 12) demonstrated a higher response compared with vancomycin (*n* = 12) (100% versus 66.7%, respectively; difference (95% CI) 33.3% (6.7%–60%).

### CABP trial

As with the ABSSSI study, clinical cure rates at test of cure (TOC) were similar across BMI categories for ceftobiprole except in the BMI ≥ 40 kg/m^2^ group in the ITT and CE populations. Cure rates for ceftobiprole in patients with a BMI of ≥40 kg/m^2^ decreased from a baseline of 76.4% to 60% and from a baseline of 86.6% to 66.7% in the ITT and CE populations, respectively (Figures 3 and 4). A decrease in clinical cure was also noted for ceftriaxone ± linezolid in the CE population in patients with a BMI of ≥40 kg/m^2^ (87.4% to 33.3%). Though no statistical differences were found when comparing ceftobiprole outcomes with baseline, the significance of these findings is uncertain due to the *post hoc* nature of the subgroup comparison, and the small number of patients considered to be severely obese. The cure rates for ceftobiprole were similar to baseline cure rates in the ITT and CE populations when evaluating patients at a BMI of <30 kg/m^2^ (75.4% and 85.1%, respectively) and ≥30 kg/m^2^ (73.3% and 86.2%, respectively). At a BMI of <35 and ≥35 kg/m^2^ in the ITT/CE populations, the ceftobiprole cure rates were 75.4%/85.5% and 69.2%/80.0%, respectively.

### Safety

Overall safety data for all three trials are summarized in Table 4.

**Table 4.** Incidence of AEs in the overall population and in BMI (kg/m^2^) subgroups from the SAB, ABSSSI and CABP Phase 3 trials

SAB	All patients (*N* = 389)		BMI < 25 kg/m^2^ (*N* = 134)		BMI 25 to <30 kg/m^2^ (*N* = 153)		BMI 30 to <40 kg/m^2^ (*N* = 86)		BMI ≥40 kg/m^2^ (*N* = 14)	
	CBP (*N* = 191)	DAP (*N *= 198)	CBP (*N* = 69)	DAP (*N* = 65)	CBP (*N* = 73)	DAP (*N *= 80)	CBP (*N* = 42)	DAP (*N* = 44)	CBP (*N* = 5)	DAP (*N* = 9)
Any TEAE	121 (63.4)	117 (59.1)	47 (68.1)	42 (64.6)	40 (54.8)	40 (50.0)	28 (66.7)	29 (65.9)	4 (80.0)	6 (66.7)
Any study drug-related TEAE	25 (13.1)	11 (5.6)	6 (8.7)	5 (7.7)	10 (13.7)	3 (3.8)	7 (16.7)	3 (6.8)	2 (40.0)	0
Any TE SAE	36 (18.8)	45 (22.7)	12 (17.4)	15 (23.1)	12 (16.4)	12 (15.0)	10 (23.8)	16 (36.4)	1 (20.0)	2 (22.2)
Any study drug-related TE SAE	2 (1.0)	4 (2.0)	1 (1.4)	1 (1.5)	0	0	1 (2.4)	3 (6.8)	0	0
Any TEAE leading to discontinuation	18 (9.4)	18 (9.1)	6 (8.7)	8 (12.3)	7 (9.6)	3 (3.8)	4 (9.5)	6 (13.6)	1 (20.0)	1 (11.1)
Any study drug-related TEAE leading to discontinuation	9 (4.7)	3 (1.5)	3 (4.3)	1 (1.5)	3 (4.1)	0	2 (4.8)	2 (4.5)	1 (20.0)	0

ABSSSI	All patients *N* = 676		BMI < 25 kg/m^2^ *N* = 250		BMI 25 to <30 kg/m^2^ *N* = 220		BMI 30 to <40 kg/m^2^ *N* = 181		BM ≥40 kg/m^2^ *N* = 24	
	CBP (*N* = 334)	VAN + ATM (*N* = 342)	CBP (*N* = 124)	VAN + ATM (*N* = 126)	CBP (*N* = 103)	VAN + ATM (*N* = 117)	CBP (*N* = 94)	VAN + ATM (*N* = 87)	BPR *N* = 12	VAN + AZM *N* = 12
Any TEAE	148 (44.3)	131 (38.6)	60 (48.4)	47 (37.3)	41 (39.8)	48 (41.0)	39 (41.5)	31 (35.6)	8 (66.7)	4 (41.7)
Any study drug-related TEAE	66 (19.8)	62 (18.1)	23 (18.5)	27 (21.4)	23 (22.3)	19 (16.2)	15 (16.0)	14 (16.1)	5 (41.7)	2 (16.7)
Any TE SAE	6 (1.8)	12 (3.5)	2 (1.6)	3 (2.4)	2 (1.9)	6 (5.1)	2 (2.1)	1 (1.1)	0	2 (16.7)
Any study drug-related TE SAE	1 (0.3)	2 (0.6)	0	1 (0.8)	0	0	1 (1.1)	0	0	1 (8.3)
Any TEAE leading to discontinuation	6 (1.8)	10 (2.9)	2 (1.6)	4 (3.2)	0	3 (2.6)	2 (2.1)	3 (3.4)	2 (16.7)	0
Any study drug-related TEAE leading to discontinuation	2 (0.6)	5 (1.5)	0	3 (2.4)	0	1 (0.9)	1 (1.1)	1 (1.1)	1 (8.3)	0

CABP	All patients (*N* = 632)		BMI < 25 kg/m^2^ (*N* = 318)		BMI 25 to <30 kg/m^2^ (*N* = 170)		BMI 30 to <40 kg/m^2^ (*N* = 74)		BMI ≥40 kg/m^2^ (*N* = 11)	
	CBP (*N* = 310)	CRO + LZD (*N *= 322)	CBP (*N* = 149)	CRO + LZD (*N* = 169)	CBP (*N* = 84)	CRO + LZD (*N* = 86)	CBP (*N* = 40)	CRO + LZD (*N* = 34)	CBP (*N* = 5)	CRO + LZD (*N* = 6)
Any TEAE	217 (70.0)	208 (64.6)	103 (69.1)	106 (62.7)	59 (70.2)	54 (62.8)	27 (67.5)	21 (61.8)	3 (60.0)	6 (100)
Any study drug-related TEAE	111 (35.8)	83 (25.8)	47 (31.5)	38 (22.5)	35 (41.7)	19 (22.1)	14 (35.0)	11 (32.4)	2 (40.0)	2 (33.3)
Any TE SAE	35 (11.3)	37 (11.5)	19 (12.8)	17 (10.1)	10 (11.9)	11 (12.8)	2 (5.0)	1 (2.9)	0	2 (33.3)
Any study drug-related TE SAE	3 (1.0)	4 (1.2)	1 (0.7)	2 (1.2)	2 (2.4)	0	0	0	0	1 (16.7)
Any TEAE leading to discontinuation	18 (5.8)	12 (3.7)	9 (6.0)	4 (2.4)	6 (7.1)	7 (8.1)	2 (5.0)	0	1 (20.0)	0
Any study drug-related TEAE leading to discontinuation	7 (2.3)	3 (0.9)	3 (2.0)	1 (0.6)	3 (3.6)	1 (1.2)	0	0	1 (20.0)	0

All values are given as *n* (%). ATM, aztreonam; CBP, ceftobiprole; CRO, ceftriaxone; DAP, daptomycin; LZD, linezolid; VAN, vancomycin.

### SAB trial

In the SAB trial, the median duration of exposure in the MITT population was 21 days for both ceftobiprole and daptomycin.^10^ The rates of treatment-emergent AEs (TEAEs) were similar between ceftobiprole and daptomycin in the overall population and across all BMI groups except the BMI ≥ 40 kg/m^2^ group (80% versus 66.7%), where the number of patients was low. Serious AEs (SAEs) were also similar in the overall population and across the BMI groups except the BMI = 30 to <40 kg/m^2^ group, where the proportion was 23.8% for ceftobiprole versus 36.4% for daptomycin. Study drug-related AEs were higher with ceftobiprole versus daptomycin in all groups except in the BMI < 25 kg/m^2^ group. Study drug-related serious AEs were similar between groups except the BMI = 30 to <40 kg/m^2^ group, where the proportion was slightly higher for daptomycin (6.8%) compared with ceftobiprole (2.4%). The rates of study drug-related SAEs remained stable as weight increased. Consistent with the known safety profile of cephalosporins, the most frequently reported treatment-related AEs were diarrhoea, nausea and vomiting across all BMI groups for ceftobiprole.

### ABSSSI trial

In the ABSSSI trial, the median duration of treatment was 6.0 days in the ceftobiprole group and 7.0 days in vancomycin + aztreonam group.^11^ Median duration of treatment with aztreonam in the comparator group was 3.0 days, with 162 (47.4%), 79 (23.1%) and 43 (12.6%) patients receiving aztreonam for more than 3, 5 and 7 days, respectively. TEAEs were reported in a higher proportion of patients treated with ceftobiprole versus vancomycin + aztreonam in the overall population and across all BMI groups except the BMI = 25 to <30 kg/m^2^ group, where the rates were similar. TE SAEs were similar across the populations except for the BMI ≥ 40 kg/m^2^ group, where it was higher for vancomycin + aztreonam though the patient numbers were small (16.7% vancomycin + aztreonam versus 0% ceftobiprole). A BMI of ≥40 kg/m^2^ was associated with more TEAEs compared with the overall population. SAEs and study drug-related SAEs were higher in the vancomycin groups compared with ceftobiprole. The most frequently reported treatment-related AEs were diarrhoea and nausea across all BMI groups for ceftobiprole.

### CABP trial

In the CABP trial, the mean (SD) study duration of exposure (CE population) was 7.2 (2.6) days for ceftobiprole and 7.8 (2.5) days for ceftriaxone ± linezolid in patients who received only IV therapy for the trial.^12^ TEAEs were reported in a higher proportion of patients treated with ceftobiprole versus ceftriaxone + linezolid in the overall population and across the different BMI groups except the BMI ≥ 40 kg/m^2^ group, where the rates were 60% with ceftobiprole and versus 100% with ceftriaxone + linezolid, though the patient numbers were low. TE SAEs were similar between the groups except in the BMI ≥ 40 kg/m^2^ group, where ceftriaxone + linezolid had a higher rate (33%) compared with ceftobiprole (0%). Study drug-related TEAEs were higher in the ceftobiprole group across the overall population and across all groups except the BMI = 30 to <40 kg/m^2^ group, where rates were similar. Study drug-related TE SAEs were low for all groups regardless of weight. The most frequently reported treatment-related AEs were diarrhoea, nausea and vomiting across all BMI groups for ceftobiprole.

## Discussion

The high prevalence of patients with obesity worldwide (16%)^1^ represents a key subset of patients seen in clinical practice. The findings of this analysis of three Phase 3 clinical trials, which included almost 24% of patients with obesity or severe obesity (BMI > 30 kg/m^2^), indicate that ceftobiprole maintains efficacy and safety in the treatment of SAB, ABSSSI and CABP in patients with obesity. Ceftobiprole at recommended dosing had similar clinical outcomes against two of the comparators that are dosed on weight (daptomycin and vancomycin), even when comparing non-standard definitions of BMI (Figure 5). There may be a signal towards decreased efficacy at higher weights (e.g. BMI ≥35 kg/m^2^) in CABP, which could indicate the challenges of dosing in obesity including adequate tissue penetration to key organs. Further investigations are warranted.

Antimicrobial dosing in the setting of obesity remains a challenge as drug distribution alterations can occur, and this patient population is often underrepresented in clinical trials. Drug distribution is a complex system affected by body composition, regional blood flow and protein binding. To further complicate this matter, renal drug clearance may be increased in obese populations due to higher glomerular surface area.^15^ A pharmacokinetic study with ceftobiprole in severely obese patients (BMI ≥ 40 kg/m^2^) (see Table S2 for summary of demographics) demonstrated lower exposures to ceftobiprole compared with non-obese patients, which was attributed to a larger volume of distribution and a higher clearance, resulting in ∼17% lower exposure (see Table S3 and Phase 1 PK and PD summary in the Supplementary data for the summary of PK and PD data). Although volume of distribution and total clearance were higher, and exposure was lower, %*fT*_>MIC_ was similar in the two groups.^9^ The lower exposure was considered unlikely to be clinically relevant and no dose adjustments based on body weight were made in the adult Phase 3 clinical studies. Further studies suggested that the decreased exposures did not affect PK/PD target attainments. As previously mentioned, the percent time above MIC (%*T*_>MIC_) of the free fraction of ceftobiprole is the primary PD driver of efficacy, and that for Gram-positive pathogens including MRSA, serum trough levels need to exceed the pathogen MIC for >30% of the dosing interval. Plasma concentrations of unbound ceftobiprole in severely obese and non-obese individuals remained above the target MIC of 4 mg/L (*fT*_>MIC_) for 76.6% and 79.7% of an 8 h dosing interval, respectively^7^ (see Table S3 for summary of PK and PD data).

Limitations of this *post hoc* analysis include low numbers of patients with obesity although this analysis included a higher percentage (23.7%) compared with the general population (16%). The lack of stratification based on BMI at randomization represents a confounder for outcomes that was not controlled. Lastly, the SAB, ABSSSI and CAP trials were not powered to detect statistical treatment differences between these subgroups, and patients enrolled in these controlled trials may not be fully representative of the real-world patients with obesity. Other special populations to evaluate going forward include patients with renal dysfunction and ARC (defined as CL_CR_ of >130 mL/min/1.73 m^2^), which can impact plasma concentrations of antimicrobials that are renally eliminated. Patients with severe obesity were noted to have higher rates of ARC compared with those without across all three studies. Further analyses including pharmacokinetic studies should be conducted to evaluate whether additional dosing schemes are warranted and whether ARC and obesity are confounders in clinical outcomes in patients with serious infections.

As obesity continues to be a health concern worldwide and expected to increase yearly, it is essential to optimize therapies to minimize morbidity and mortality. Different pharmacokinetic studies suggest a need for an increased dose of β-lactams in obese patients, mainly due to an increase in the volume of distribution and renal clearance.^2^ This analysis of over 800 patients, including 197 patients with obesity or severe obesity, from three Phase 3 trials suggests the efficacy and safety of ceftobiprole are maintained in patients with obesity without the need for dose adjustment. Further studies to confirm these preliminary findings are necessary. Future clinical Phase 3 studies with new antimicrobials should consider expanding recruitment efforts for obese patients and evaluating key pharmacokinetic parameters to determine the impact of altered drug distribution and clearance in this special population.

## Supplementary Material

dkaf096_Supplementary_Data

## Transparency declarations

T.L.H. has served as an investigator for the SAB trial, received funds for speaking at a Basilea Investor Event, and is a member of the Basilea advisory board for ceftobiprole. A.F.S has served as a consultant for Basilea. J.S.O. served as an investigator for the ABSSSI trial. V.G.F. has served as an investigator for the SAB trial, is a paid consultant for Basilea, and received Grant/Research Support from Basilea. M.E., M.J., D.I., K.L. and M.S. are employees of Basilea Pharmaceutica International who may own stock and/or hold stop stock options in Basilea Pharmaceutica International in Allschwil, Switzerland. The Phase 3 studies were funded by Basilea Pharmaceutica International (Allschwil, Switzerland). Medical writing and/or editorial assistance was provided by Hina N. Patel, PharmD of Scientific and Medical Affairs Consulting, LLC, East Greenville, PA, USA

## Supplementary data

Tables S1 to S3 are available as Supplementary data at *JAC* Online.
