# Supplementary material for: Impact of obesity on clinical outcomes in patients treated with ceftobiprole: results from Phase 3 clinical trials
Source: J Antimicrob Chemother. 2025 Mar 28;80(6):1526–34. doi: 10.1093/jac/dkaf096 (PMC12129573; doi:10.1093/jac/dkaf096)
Supplement: dkaf096_Supplementary_Data [file dkaf096_supplementary_data.docx]

**Supplementary Appendix**

This appendix has been provided by the authors to give readers additional information about their work

Supplement to: Holland, et al. Impact of obesity on clinical outcomes in patients treated with ceftobiprole from Phase 3 clinical trials.

**Table of Contents**

**Supplementary Background on Clinical Trials**

Table S1. Key Design Elements of Phase 3 clinical trials

Definitions of Analysis Populations

**Supplementary Results of Phase 1 pharmacokinetic and pharmacodynamic study in severely obese patients**

Table S2. Phase 1 Study Subject Demographics

Table S3. Pharmacokinetic and pharmacodynamic parameters of plasma ceftobiprole in seveerly obese compared to non-obese patients

Phase 1 Pharmacokinetic and Pharmacodynamic Summary

**Supplementary References**

**Supplementary Background on Clinical Trials**

**TABLE S1.** Key Design Elements of Phase 3 Clinical Trials

| Parameter | **SAB^1^** | **ABSSSI^2^** | **CABP^3^** |
| --- | --- | --- | --- |
| Design | Randomized, double-blind, active-controlled, parallel-group, multicenter between ceftobiprole and  daptomycin | active-controlled, parallel-group,  multicenter between ceftobiprole and vancomycin plus aztreonam | Randomized, double-blind, active-controlled,  parallel-group, multicenter between ceftobiprole and  ceftriaxone ± linezolid |
| Treatment Groups | Ceftobiprole  **ITT** N = 192  **SAF** N = 191  Comparator  **ITT** N = 198  **SAF** N = 198 | Ceftobiprole  **ITT** N = 335  **SAF** N = 334  Comparator  **ITT** N = 344  **SAF** N = 342 | Ceftobiprole  **ITT** N = 314  **SAF** N = 310  Comparator  **ITT** N = 324  **SAF** N = 322 |
| Ceftobiprole dose | Day 1 to Day 8  500 mg every 6 h  Day 9 onwards  500 mg every 8 h | 500 mg every 8 h  CLCR 30 to ≤ 50 mL/min  500 mg every 12 h  CLCR >10 to ≤ 30 mL/min  250 mg every 12 h | 500 mg every 8 h  CLCR 30 to ≤ 50 mL/min  500 mg every 12 h  CLCR >10 to ≤ 30 mL/min  250 mg every 12 h |
| Comparator dose | 6 mg/kg every 24 h with the option to use doses up to 10 mg per kilogram, if consistent with institutional practice | 1,000 mg (or 15 mg/kg)  every 12 h  Aztreonam 1,000 mg every 12 h | 2 g every 24 h  If required, linezolid 600 mg every 12 h |
| Duration of treatment | 21 to 42 days with duration determined by the site investigators. | 5–10 days with a possible extension to 14 days if clinically required. | 7 days, but this could be extended to 14 days at the investigator’s discretion |
| Patient population | Adult patients with SAB  considered complicated and treated with effective  antibacterial treatment for less than 48 h | Adult patients with ABSSSI  (cellulitis/erysipelas, major cutaneous abscess, wound infection) requiring I.V. antibiotic treatment | Patients hospitalized with  CABP requiring treatment  with I.V. antibiotics for at  least 3 days |
| Primary efficacy endpoint | Overall success at PTE as  assessed by the DRC (i.e. 70 days after randomization) | Early clinical response 48–72 h after start of treatment | Clinical cure rate at TOC |
| Primary efficacy  analysis population (FDA) endpoint | Modified ITT  Ceftobiprole N = 189  Comparator N = 198 | ITT  Ceftobiprole N = 335  Comparator N = 344 | ITT / CE  Ceftobiprole N = 314/ 231  Comparator N = 324/238 |

ABSSSI = acute bacterial skin and skin structure infections; AE = adverse event; CABP = community-acquired bacterial pneumonia; CE = Clinically Evaluable; CLCR = creatinine clearance;

DRC = Data Review Committee; EOT = end-of-treatment; HABP = hospital-acquired bacterial pneumonia, ITT = Intent-to-Treat; I.V. = intravenous; LFU = late/last follow-up; PTE = posttreatment

evaluation; SAB = *Staphylococcus aureus* bacteremia; SAF = Safety; TOC = test-of-cure.

**Definitions of Analysis Populations**

Clinically evaluable (CE) population: included all treated patients with a diagnosis of CAP, except those who received <48 h of study drug or a non-study antibiotic with activity against CAP pathogens, received <80% of the intended doses of study drug, were cured with <5 days of therapy, had a pathogen(s) resistant to either study regimen, did not have a pulmonary infiltrate confirmed by central radiology, died prior to TOC from a cause unrelated to pneumonia, were missing a TOC visit, or had positive baseline IgM serology for *M. pneumoniae or C. pneumoniae (*and no typical bacterial pathogen identified).^3^

Intent-to-treat (ITT) population: Comprised of all randomized patients.^,2,3^

Modified Intent to Treat (MITT) population: included all the patients who had undergone randomization and had received at least one dose of ceftobiprole or daptomycin and who had a confirmed baseline blood culture growing *S. aureus*.^1^

Supplementary Results of Phase 1 pharmacokinetic and pharmacodynamic study in severely obese patients

**TABLE S2.** Phase 1 study subject demographics^4^

|  | Severely obese (N=12)  BMI ≥40 kg/m^2^ | Non-obese (N=13)  BMI 18–30 kg/m^2^ |
| --- | --- | --- |
| Sex (M:F) | 4:8 | 4:9 |
| Age (years)  Mean (SD)  Median (min–max) | 43.0 (27–52)  41.1 (7.4) | 38.0 (19–55)  38.8 (10.5) |
| Weight (kg)   Mean (SD)  Median (min–max) | 130.9 (17.9) 129.3 (100–163) | 73.5 (11.0) 72.6 (50–91) |
| BMI (kg/m^2^)  Mean (SD)  Median (min–max) | 45.5 (4.3) 44.0 (41–54) | 24.0 (2.5) 23.7 (19–30) |

BMI, body mass index; SD, standard deviation; M, male; F, female

**Table S3.** Pharmacokinetic and pharmacodynamic parameters of plasma ceftobiprole in severely obese compared to non-obese patients^4^

|  | Severely obese (N=11^a^)  BMI ≥40 kg/m^2^ | Non-obese (N=13)  BMI 18–30 kg/m^2^ |
| --- | --- | --- |
| C_max_, µg/mL | 21.4 ± 3.0 | 30.2 ± 4.3 |
| AUC_∞_, µg∙h/mL | 91.0 ± 11.7 | 110 ± 20.1^b^ |
| t_½_, h | 3.4 ± 0.3 | 3.2 ± 0.5^b^ |
| V_ds,_ L | 27.2 ± 3.9 | 21.6 ± 5.1^b^ |
| CL, L/h | 5.6 ± 0.7 | 4.7 ± 0.7^b^ |
| %fT>MIC (4 mg/L)^c^ | 76.6 ± 9.2^c^ | 79.7 ± 7.3 |

^a^One subject who received treatment was excluded from pharmacokinetic analysis
^b^N =12
^c^8-h dosing interval; data are mean ± SD
C_max_, maximum plasma concentration; AUC, area under the plasma concentration-time curve; t_1/2_, elimination half-life; V_ds_, volume of distribution; CL, total clearance

**Phase 1 Pharmacokinetic and Pharmacodynamic Summary^4^**

Individuals received a single IV infusion of ceftobiprole 500 mg over 2 hours. In severely obese subjects, the total clearance (CL) of ceftobiprole (Table S3) was 19.1% higher than in non-obese individuals. The volume of distribution (V_ds_) of ceftobiprole (Table S3) was 25.9% higher than in non-obese individuals. There were no obvious correlations between CL and V_ds_ with BMI or weight. Though exposure (C_max_ and AUC_∞_) of ceftobiprole was lower in severely obese than in non-obese individuals, the percent time of free drug above the minimum inhibitory concentration was similar between the two groups. Based on this data, it is recommended that dose adjustment of ceftobiprole is not required for severely obese patients.

**Supplementary References**

1. Holland TL, Cosgrove SE, Doernberg SB, et al; ERADICATE Study Group. Ceftobiprole for treatment of complicated *Staphylococcus aureus* bacteremia*. N Engl J Med* 2023; **389**: 1390-1401.
2. Overcash JS, Kim C, Keech R, et al. Ceftobiprole compared with vancomycin plus aztreonam in the treatment of acute bacterial skin and skin structure infections: results of a Phase 3, randomized, double-blind trial (TARGET). *Clin Infect Dis* 2021; **73**: e1507-17.
3. Nicholson SC, Welte T, File TM Jr, et al. A randomised, double-blind trial comparing ceftobiprole medocaril with ceftriaxone with or without linezolid for the treatment of patients with community-acquired pneumonia requiring hospitalisation. *Int J Antimicrob Agents* 2012; **39**: 240-6.
4. Schmitt-Hoffmann A, Engelhardt M, Spickermann M, et al. Pharmacokinetics and pharmacodynamics of ceftobiprole in adults who are severely obese. *Twenty-sixth European Congress of Clinical Microbiology and Infectious Diseases*, *Amsterdam, the Netherlands, 2016.* Abstract P1250.
